# Accurate identification of clinically important *Acinetobacter* spp.: an update

**DOI:** 10.2144/fsoa-2018-0127

**Published:** 2019-06-27

**Authors:** Saranya Vijayakumar, Indranil Biswas, Balaji Veeraraghavan

**Affiliations:** 1 Department of Clinical Microbiology, Christian Medical College, Vellore 632 004, Tamil Nadu, India; 2 Department of Microbiology, Molecular Genetics & Immunology, University of Kansas Medical Center, Kansas City, KS 66160, USA

**Keywords:** ACB complex, *Acinetobacter baumannii*, MALDI-TOF, matrix-assisted laser desorption ionization-time of flight mass spectrometry, molecular methods, phenotypic methods

## Abstract

*Acinetobacter* species have emerged as one of the most clinically important pathogens. The phenotypic techniques which are currently available are insufficient in accurately identifying and differentiating the closely related and clinically important *Acinetobacter* species. Here, we discuss the advantages and limitations of the conventional phenotypic methods, automated identification systems, molecular methods and MALDI-TOF in the precise identification and differentiation of *Acinetobacter* species. More specifically, several species of this genus are increasingly reported to be of high clinical importance. Molecular characterization such as of *bla*_OXA-51_-like PCR together with *rpoB* sequencing has high discriminatory power over the conventional methods for *Acinetobacter* species identification, especially within the *Acinetobacter calcoaceticus–Acinetobacter baumannii* complex.

## Lay abstract

*Acinetobacter* species are considered to be one of the most important pathogens and associated with increased mortality. The species within the *Acinetobacter calcoaceticus–Acinetobacter baumannii* complex have emerged as high priority pathogens, especially in intensive care units, thereby posing a challenge to infection management practices. However, identification of *Acinetobacter* to the species level is difficult. Clear differentiation among various *Acinetobacter* species with available standard biochemical methods and automated systems is challenging. Although various molecular methods are available, they are not regularly used in diagnostic laboratories. The advantages and disadvantages of different methods useful in the accurate identification of *Acinetobacter* species are discussed in this review.

The genus *Acinetobacter* includes a heterogeneous group of bacteria that have been considered ‘the most troublesome’ by clinicians and scientists [1]. This genus has undergone substantial taxonomic modification. Classical phenotypic methods like biochemical tests have proven to be difficult at the species-level identification of *Acinetobacter* [2].

Currently, the genus *Acinetobacter* comprises 59 different species with distinct names (www.bacterio.net/acinetobacter.html; last accessed on October 2018). A total of 11 effectively but not validly published named species and nearly 15 tentative species with designation have been reported (http://apps.szu.cz/anemec/anemec.html) [3].

Initially, the *Acinetobacter calcoaceticus–Acinetobacter baumannii* complex (ACB complex) comprised four species: *Acinetobacter calcoaceticus* (genomic species 1)*, Acinetobacter baumannii* (genomic species 2), *Acinetobacter* pittii (previously named as genomic species 3) and *Acinetobacter nosocomialis* (previously named as genomic species 13 TU). Recently, two new species, *Acinetobacter seifertii* (previously known as genomic species close to 13TU) and *Acinetobacter dijkshoorniae* (closely related to *A. pittii*) were also included within the ACB complex [4,5].

Currently, five *Acinetobacter* species associated with human diseases (*A. baumannii*, *A. pittii*, *A. nosocomialis*, *A. seifertti* and *A. dijkshoorniae*) and one environmental *Acinetobacter* species (*A. calcoaceticus*) are very closely related and display similar phenotypic and biochemical properties; therefore, it is difficult to distinguish them at the individual species level [6]. The accurate identification of the clinically important members of this group is only possible by molecular methods. Among the ACB complex, *A. baumannii* is the most important clinically relevant species, responsible for causing 80% of infections [7,8]. *A. pittii* and *A. nosocomialis* are also considered clinically important and have been implicated in causing community and hospital-acquired infections [4,5,8]. The two novel species mentioned previously (*A. seifertii* and *A. dijkshoorniae*) were also isolated from human clinical specimens [4,5]. *A. calcoaceticus* is considered to be a nonpathogenic environmental organism isolated from soil and is rarely involved in causing diseases [4,5].

*Acinetobacter* species were found to be associated with increased mortality [9]. The ACB complex has emerged as a high priority nosocomial pathogen, especially in intensive care units, thereby posing a challenge to infection management practices [10].

However, identification of *Acinetobacter* to the species level remains complicated. Clear differentiation among various *Acinetobacter* species using standard biochemical methods and automated systems are challenging, thus leading to inaccurate identification [9]. Although various molecular methods are available, they are not regularly employed in routine diagnostic laboratories [11].

Accurate identification of *Acinetobacter* is essential as these nosocomial pathogens, *A. baumannii*, *A. pittii* and *A. nosocomialis*, differ in their biological and pathological characteristics [12,13]. In addition, differences have also been observed among these pathogens in their ability to colonize the skin, antimicrobial susceptibility and mechanisms of antimicrobial resistance [12,13]. *A. baumannii* is more predominantly isolated from patients with poor outcome and increased resistance to antimicrobial agents when compared with other pathogens within the ACB complex [12,13]. *A. baumannii* is responsible for causing bacteremia, ventilator-associated pneumonia, urinary tract infections and skin and soft tissue infections. In 2017, Liu *et al.* reported *A. baumannii* to have a high mortality rate, of 29.8–36.9% [14]. Jain *et al*. reported that *A. pittii* as the most frequently isolated species with a lower rate of mortality in children [9]. Recent studies from other countries have shown that *A. pittii* and *A. nosocomialis* were responsible for 29% of ACB complex bacteremia in the USA, 24–45% in Taiwan, 50% in Korea and 66% in Norway [14].

A study by Lee *et al*. in 2013 clearly demonstrates important differences in the clinical features and outcome of pneumonia caused by *A. baumannii* and *A. nosocomialis* [15]. Patients infected with *A. baumannii* were more likely to have severe illness, increased antimicrobial resistance and higher mortality when compared with *A. nosocomialis*, supporting the concept that the pneumonia caused by *A. baumannii* and *A. nosocomialis* should be considered as two different clinical entities [15].

Therefore, it is important to have species-level identification of the ACB complex due to differences in epidemiology, antimicrobial resistance patterns and clinical outcomes, and reporting of infection from the ACB complex without further speciation may be considered inadequate, leading to inferior clinical care and inappropriate use of antibiotics [16].

## Role of phenotypic methods

The identification scheme for *Acinetobacter* species, first proposed in 1986 by Bouvet and Grimont, was based on 28 phenotypic tests – physiological, nutritional and enzymatic tests [17]. Further refinement came in 1987, which includes growth at different temperatures (37, 41 and 44°C), gelatin hydrolysis, assimilation of 14 carbon sources and acid production from glucose [18]. Based on the growth characteristics of *Acinetobacter* spp. at different temperatures, the scheme facilitated the differentiation of most clinically relevant isolates as *A. baumannii* , *A. nosocomialis*, *A. pitti* and *A. calcoaceticus*. [18,19].

The species-level identification of *Acinetobacter* is not consistent and reliable with the existing phenotypic methods [6]. Recently, the extension of the Bouvet and Grimont phenotypic system was proposed by Nemec, and includes growth at different temperatures (32, 35, 41 and 44°C), glucose acidification, hemolysis, gelatinase production and growth on 36 single carbon and energy sources (http://apps.szu.cz/anemec/anemec.htm). A list of phenotypic tests for the identification of ACB complex is detailed in Table 1.

**Table 1.** Phenotypic characterization of species within *Acinetobacter calcoaceticus–Acinetobacter baumannii* complex.

Characteristic	*Acinetobacter calcoaceticus* [3]	*Acinetobacter baumannii* [3]	*Acinetobacter pittii* [3]	*Acinetobacter nosocomialis* [3]	*Acinetobacter seifertii* [4]	*Acinetobacter dijkshoorniae* [5]
Glucose acidification	−	+	V (+)	+	+	+
**Assimilation of:**						
Adipate	+	V (+)	+	V (+)	V (+)	+
β-alanine	V (−)	+	V (+)	V (+)	V (+)	+
L-arabinose	V (−)	V (+)	V (+)	+	−	+
Azelate	+	V (+)	+	V (+)	V (+)	+
Citroconate	−	V (+)	−	−	−	−
Gentisate	−	V (−)	V (−)	V (−)	V (+)	+
Leucine	V (−)	V (+)	V (+)	V (+)	V (+)	+
Malate	D	V (+)	V (+)	+	V (+)	+
Ornithine	+	V (−)	V (+)	V (+)	V (+)	V (+)
Putrescine	+	V (+)	+	V (+)	V (+)	+
Ribose	V (−)	V (+)	V (−)	V (+)	−	V (+)
Tartrate	V(−)	V (−)	V (+)	−	V (+)	V (+)
Trigonelline	V (−)	V (+)	V (+)	V (−)	−	V (+)

D: Doubtful; V (+): Variable, some strains are positive; V (−): Variable, some strains are negative.

## Role of automated bacterial identification systems

Various commercial automated systems for identification are available and widely used in clinical microbiology laboratories [20,21]. These automated systems are based on different technologies such as measurement of growth of the microorganisms using biochemical or physical parameters, CO_2_ production, color, turbidity, changes in fluorescence due to microbial consumption of biochemical and carbohydrate substrates in media, production or consumption of heat, changes in pressure due to microbial consumption and bacterial detection by viability staining and metabolic activity [20,21]. The commonly used automated commercial systems for the species-level identification of *Acinetobacter* in the clinical laboratories includes API 20NE, VITEK 2, Phoenix, Biolog, MicroScan WalkAway and Accelerate Pheno™ system [18].

The API 20NE is available with microtubes and has dehydrated media and substrates. The end products of the test are detected by an indicator or addition of reagents. The API 20NE, manual method from bioMerieux compared with conventional biochemicals in the identification of 198 isolates has reported an overall agreement of 92% [19,20]. With the updated database of API 20NE (version 5.1), Bernards *et al*. achieved 87% accurate identification [2]. The Biolog system for automated identification of bacteria is based on the redox system [22]. Bernard and his colleagues compared strain groupings of *Acinetobacter* by Biolog cluster analysis with DNA–DNA hybridization (DDH) studies and found 84.5% of the strains were correctly identified at the genus level by the Biolog system [23].

The BD Phoenix system involves the use of an oxidation–reduction indicator and turbidometric growth detection, whereas the MicroScan WalkAway system uses photometer or fluorometer to determine the growth development. The VITEK 2 system utilizes the growth based technology for microbial identification. Wang and colleagues have reported that VITEK MS method was able to accurately identify three species (*A. junii, A. hemolyticus* and *A. johnsonii*) but failed to discriminate the species within the ACB complex [24]. Such results are expected because the clinical identification system depends on the database already exists in the automated systems that are not comprehensive [18]. In addition, the substrates used for species identification have not been designed specifically for the identification of *Acinetobacter* [18].

Other the US FDA-cleared automated identification systems designed to identify the organisms directly from positive blood culture bottles include the Verigene system (Nanosphere) gram negative blood culture (BC-GN) nucleic acid tests and the FilmArray blood culture identification panel (Biofire diagnostics) [25,26]. The Verigene system involves purification of nucleic acids and hybridization to oligonucleotide gold nanoparticles on microarray [21]. In a study by Kim *et al*., Gram-negative bacteria were cultured from 150 samples, of which 22 were *Acinetobacter* spp and all were accurately identified by the Verigene [26]. The filmArray identification system involves extraction of DNA followed by PCR and melt curve analysis [21]. Phenotypically, *A. baumannii* was identified as a member of the ACB complex followed by bidirectional sequencing of the 16S rRNA gene. After comparison, the FilmArray blood culture identification detected all the 51 culture isolates as *A. baumannii* [27].

The Accelerate Pheno system, a fully automated system, has the capability of performing identification and antimicrobial susceptibility testing directly from positive blood cultures [28]. The Accelerate Pheno system combines gel electrofiltration and a broad panel of fluorescence *in situ* hybridization for bacterial identification and morphokinetic cellular analysis, which measures the response of individual cells and colonies to antibiotics over time using time-lapse imaging for antimicrobial susceptibility testing [29]. The overall identification performance has been reported with the sensitivity level of 98.6% and specificity of 99.7% with a positive predictive value and a negative predictive value of 100% for *A. baumannii* [29].

Snyder and colleagues have compared the BD Phoenix system with MicroScan WalkAway and showed 100% concordance for the identification (ID) of nonfermentative Gram-negative bacilli (NFGNB) [30].

## Molecular identification of *Acinetobacter* species

For the molecular identification of *Acinetobacter* species, several methods have been developed, studied and shown to be adequate. This includes DNA–DNA hybridization (DDH), DNA sequence-based analysis and restriction analysis of PCR amplified DNA sequences (PCR-restriction fragment length polymorphism [PCR-RFLP]). The following sections briefly describe each of the methods.

## DNA–DNA hybridization

DDH is a gold standard method where the whole genome of the organisms is compared for delineation of bacterial species. Johnson and colleagues assigned *Acinetobacter* strains to five distinct DNA groups with four strains ungrouped [31]. Bouvet and Grimont distinguished 12 genospecies [32]. Bouvet and Jeanjean described genomic species 13–17 [33]. A parallel study to Bouvet and Jeanjean describing three new genomic species 13–15 was reported by Tjernberg and colleagues [34]. In 1988, the genomic sp. 12 was described as *A. radioresistens* [35].

Although DDH has been considered a gold standard for bacterial species differentiation over the past 50 years, due to several disadvantages like a labor-intensive and error prone nature, genotype-based technology was needed [36]. As whole-genome sequencing is available, numerous overall genome relatedness indices (OGRI) were developed to replace DDH [37].

The OGRI is digital DDH-like similarity indices based on computational comparisons of two genome sequences [36]. Various OGRI are available, including average nucleotide identity (ANI), genome BLAST distance phylogeny and maximal unique matches index [36]. Among these, ANI is most widely used and considered as the gold standard method for species demarcation [36,37]. Generally, ANI values of 95–96% are accepted for species delineation, corresponding to the traditional 70% DDH threshold [36]. To calculate the reciprocal ANI values, a new algorithm called OrthoANI (ANI by orthology) is available [37].

Chan *et al*., using the genus *Acinetobacter*, examined whether bacterial taxonomy could rely upon genome-based sequence data using ANI [38]. For example, *A. nosocomialis* NCTC 10304 exhibits ANI values more than 95% with 14 sequenced *A. baumannii*, thereby confirming the designation as *A. baumannii* NCTC 10304. Similarly, *A. calcoaceticus* PHEA-2 was designated as *A. pittii* PHEA-2 [38]. Since then various studies have used ANI for species delineation of the genus *Acinetobacter* [4,5,39,40].

## DNA sequence-based analysis

DNA sequence-based analysis is used to understand the relatedness of organisms from their DNA sequence similarity. Various DNA sequences like 16S rRNA, *rpoB*, 16S–23S intergenic spacer (ITS) have been studied in classification and identification of *Acinetobacter*.

## 16S rRNA & *rpoB* sequencing

16S rRNA sequencing is the most commonly used gold standard technique for unknown identification of bacterial species [41]. The 16S rRNA gene of bacteria contains nine hypervariable regions that show sequence diversity among the different bacterial species and are therefore used for species-level identification [42]. The V8 hypervariable region of 16S rRNA is useful in the identification of *Acinetobacter* at the genus level [43]. Misbah *et al*. amplified 1500 bp 16S rRNA region using three primer pairs to identify clinical isolates of *Acinetobacter* species and all were identified as *A. baumannii* [44]. The intraspecies similarity of 16S rRNA for *A. baumannii* is 99.9–100%, *A. nosocomialis* 99.7–100%, *A. pittii* 99.8–100%, *A. calcoaceticus* 97.6–99.3%, *A. seifertii* 97.4–97.8%, *A. dijkshoorniae* 99–100%. The interspecies similarity of 16S rRNA of *A. baumannii* with *A. nosocomialis, A. pittii, A. calcoaceticus, A. seifertii* and *A. dijkshoorniae* were 97.2, 97.1, 96.9, 98.5 and 97.1%, respectively. Although 16S rRNA sequencing was reported to be polymorphic for the identification of other bacterial genera, it is not effective in differentiating *Acinetobacter* at the species level due to its extremely low polymorphic nature of the variable region [45]. Due to this limitation, protein encoding genes like *rpoB, gyrB* and *recA*, which evolve much faster, were investigated as diagnostic biomarkers. The *rpoB* gene is 4089 base pair (bp) long and only one copy is present [45]. The *rpoB* gene has four variable regions which includes two polymorphic regions and two flanking spacers enclosed by conserved sequences [45]. The intraspecies similarity of *rpoB* for *A. baumannii* was 98.7–100%, *A. nosocomialis* 98.7–100%, *A. pittii* 98.6–100%, *A. seifertii* 98.5–100%, *A. dijkshoorniae* 98.6–100% and *A. calcoaceticus* 97.7–99.7% [5]. The interspecies similarity of *rpoB* for *A. baumannii* with *A. pittii, A.nosocomialis, A. calcoaceticus, A. seifertii* and *A, dijkshoorniae* were 92–93%, 94–95%, 91–92%, 94–95% and 93–94%, respectively [5].

Recently, Lee and colleagues reported, 93.4 and 98.2% accuracy for the species-level identification of *Acinetobacter* by 16S rRNA and *rpoB* sequencing, respectively [46]. Similarly, Khosravi’s group revealed that 16S rRNA sequencing of *A. grimontii* and *A. junii*, was ambiguous due to high similarity between these two species, whereas the *rpoB* gene sequencing showed 100 and 99% similarity to *A. grimontii* and *A. junii* respectively, [47]. Initially, Vaneechoutte *et al*. proposed that *A. grimontii* as a heterotypic synonym of *A. junii* by DDH and AFLP [48]. Wang *et al.* reported that the similarity of *rpoB* sequences of *A. grimontii* and *A. junii* is greater than 99%, thereby suggesting their combination into one species [49].

La Scola’s group investigated the efficacy of *rpoB* gene sequencing for the differentiation and identification of 17 reference strains of *Acinetobacter* spp. and seven unnamed genome species. The percentage similarities of *rpoB* gene sequences of different strains varied between 83 and 99% [45]. Furthermore, Gundi and colleague studied the use of partial *rpoB* sequence analysis (PRBS) on a collection of *Acinetobacter* spp. by analyzing residues spanning the 350 base pair (bp) hypervariable zone of the *rpoB* gene [50]. With partial *rpoB* sequence analysis, it was found that while interspecies similarity were well separated (ranging from 88.3 to 96.9%), the intraspecies similarity of the *rpoB* gene sequences was found to be 98–100% for *A. baumannii*, 98–100% for *A. nosocomialis*, 98–100% for *A. pitti* and 98.6–100% for *A. calcoaceticus* [50].

In summary, while 16S rRNA sequencing is highly reliable at the genus level, it has poor discriminatory ability at the species level. In contrast, *rpoB* genes among *Acinetobacter* spp. are highly variable and considered to be appropriate for species identification [49].

## Restriction analysis of PCR amplified DNA sequences

This method involves amplification and restriction of conserved gene sequences using various restriction enzymes. Several gene sequences such as 16S rRNA, 16S–23S ITS and *recA* are widely used [51].

In the amplified ribosomal DNA restriction analysis (ARDRA) method, amplification of the 16S rRNA gene followed by RFLP using different restriction enzymes enabled identification of all *Acinetobacter* species except *A. haemolyticus* (genomic species 4) and *A. johnsonii* (genomic species 7) [1]. However, when Nowak *et al*. used the 16S–23S ITS region, identical PCR and RFLP patterns were observed [52]. Multiplex PCR targeting *recA* and 16S rDNA genes followed by RFLP yielded reliable identification of *Acinetobacter* geno species five, seven and ten including the closely related species within ACB complex [53].

Although the above methods are helpful in understanding the clinical relevance and epidemiology of *Acinetobacter* species, they are too laborious for day-to-day application in the laboratory or field setting. As these techniques involve skilled labor, they are restricted only to reference laboratories.

## Sequence-based analysis of other gene targets

The other sequence targets available for the identification and discrimination of *Acinetobacter* species are summarized in Table 2. A multiplex PCR targeting the *recA* and 16S–23S rRNA ITS region for the identification of *Acinetobacter* species within the ACB complex was developed using 22 reference strains and 138 clinical isolates of *Acinetobacter* spp. The multiplex PCR was found to be 100% accurate in identification of *Acinetobacter* at the species level, which was later confirmed by sequence analysis [54]. In 2013, Soo and colleagues developed a loop-mediated isothermal amplification (LAMP) method for identification of *A. baumannii* using the 16S–23S rRNA ITS region. The sensitivity of the LAMP assay was found to be 100-fold superior to the simple multiplex PCR method in identifying *Acinetobacter* species [55]. However, the main drawback is that the LAMP method has not been studied on different clinical isolates.

**Table 2.** Various PCR-based targets for identification of *Acinetobacter* spp.

Target	Number of specimens tested	Molecular technique	Accuracy rate	Study (year)
16S rRNA and *rpoB*	124 strains of *nonbaumanni*i group and 52 strains of *A. baumannii*	PCR and sequencing	• 98.2% for *rpoB* 93.4% for 16S rRNA sequencing at species level, respectively	Lee *et al*. (2014) [46]
	197 clinical isolates of *Acinetobacter* spp.	PCR–RFLP and sequencing	• Simultaneous analysis of 16S rRNA and *rpoB* enabled identification of *Acinetobacter* up to species level	Khosravi *et al*. (2015) [47]
16S-23S rRNA ITS and *recA*	28 reference strains and 79 clinical isolates	PCR and sequencing (16S–23S ITS region)	• Identification rate – 96.2% • Reliable and promising tool for identification of species within ACB complex	Chang *et al*. (2005) [74]
	138 clinical isolates	PCR (16S–23S ITS and *recA*)	• All *Acinetobacter* species positive for *recA* gene • Only *A. baumannii* species positive for 16S–23S ITS gene	Chen *et al*. (2007) [54]
	84 *Acinetobacter* spp and 21 non-*Acinetobacter* spp.	LAMP (16S–23S ITS region)	• 100% accuracy in identification of isolates & 100-fold sensitive than conventional PCR • Suitable for detection of *A. baumannii* from clinical or environmental samples	Soo *et al*. (2013) [55]
	400 carbapenem resistant *Acinetobacter* spp.	Multiplex real time PCR (16S-23S ITS and *recA*)	• 100% concordance results with conventional PCR • High sensitivity and specificity rate (100%) than conventional PCR	Yang *et al*. (2016) [75]
*gyrB*	124 strains of *nonbaumannii* group and 52 strains of *A. baumannii*	PCR and sequencing (*gyrB*)	• 77.2 and 100% accuracy rate and concordance, respectively, at species level	Lee *et al*. (2014) [46]
	118 clinical isolates (31 *A. baumannii* and 54 *A. nosocomialis*)	PCR	• 100% accurate in distinguishing *A. baumannii* and *A. nosocomialis*	Higgins *et al*. (2007) [62]
*bla*OXA-51_like_	170 isolates	PCR (*bla*OXA-51_like_)	• 141 isolates were positive, confirming them as *A. baumannii* and absent in non-*baumannii* isolates	Turton *et al*. (2006) [56]

ITS: Intergenic spacer; PCR: Polymerase chain reaction; PCR–RFLP: PCR–restriction fragment length polymorphism.

Another reliable marker for identification of *A. baumannii* is the *bla*_OXA-51_-like gene, which is intrinsic to *A. baumannii*. The utility of *bla*_OXA-51_-like gene-specific PCR has been studied by Turton and colleagues on 106 *A. baumannii* isolates and 23 different *Acinetobacter* spp, which were previously identified by amplified 16S rDNA restriction analysis (ARDRA). All 106 *A. baumannii* clinical isolates, as well as the ATCC 19606 strain, flagged positive for *bla*_OXA-51_-like gene, whereas isolates that showed negative were identified as another species of *Acinetobacter.* So far, 95 variants of *bla*_OXA-51-_like gene have been described and the possible limitation of this PCR method could be due to its inability to detect all the described variants of the *bla*_OXA-51_ gene [56,57].

However, false-positive and false-negative results for detection of the *bla*_OXA-51_ gene were reported by a few studies, as mentioned below. False-positive results were observed by Chen’s group in two different studies involving 676 and 74 isolates of non-*baumannii Acinetobacter* species, which showed 0.9 and 5.5% prevalence of the *bla*_OXA-51_-like gene, respectively. The *bla*_OXA-51_-like gene has weak carbapenem hydrolyzing activity. Insertion sequences (ISs) such as IS*Aba1* could be inserted upstream, leading to overexpression of the bla_OXA-51_-like genes [58]. It was reported that there is presence of the *bla*_OXA-51_-like gene in non*-baumannii* species including *A. nosocomialis* and other genomic species close to *A. nosocomialis.* All these isolates contain the upstream insertion of IS*Aba1* on plasmids, which might have been transferred from *A. baumannii* by the horizontal gene transfer method [59].

The false negative issue was also observed during an outbreak situation, where approximately 14 isolates generated a 1.7-kb PCR product instead of 353 bp for this marker. Sequencing of the 1.7-kb PCR band revealed disruption of the *bla*_OXA-51_-like gene by the insertion element IS*Aba19*, thereby suggesting the *bla*_OXA-51_-like gene to be an unsuitable molecular marker for *A. baumannii* identification [60]. A separate study also reported disruption of the *bla*_OXA-51_-like gene by insertion elements like IS*Aba15* and IS*Aba19* [61].

Notably, presence of such plasmid-borne insertion elements along with *bla*_OXA-51_-like gene confers high level carbapenem resistance, thereby limiting the use of *bla*_OXA-51_-like gene as a target for differentiating *A. baumannii* from other *Acinetobacter* species. Therefore, it is essential to include additional gene targets like *rpoB* sequencing along with the *bla*_OXA-51_-like PCR for the differentiation of *A. baumannii* from ACB complex.

Multiplex PCR based on species-specific genes is considered simple, rapid and reliable for identification of different species within the ACB complex. This multiplex PCR is robust, reproducible and cost effective and yields results in less than 2 h. A multiplex PCR based on *gyrB* primers was proposed for the identification of *A. baumannii* and *A. nosocomialis* [62]. This multiplex PCR was expanded to include identification of *A. pittii* and *A. calcoaceticus* [63]. A total of 495 clinical isolates of *Acinetobacter* were subjected to *gyrB* multiplex PCR and VITEK2 analysis. The accuracy rates were found to be 90.5 and 76.6% for *gyrB* multiplex PCR and VITEK2, respectively [46]. The *gyrB* multiplex PCR showed 100% concordance in identifying species within the ACB complex whereas VITEK2 was able to identify only *A. baumannii* and failed to identify other species within the ACB complex [46].

Although various target regions like *recA* and *gyrB* are available for the identification and differentiation of *Acinetobacter* species within the ACB complex, none of them has been validated for the recently included novel species *A. seifertii* and *A. dijkshoorniae*, which can be considered as a limitation.

## Role of MALDI-TOF

Matrix-assisted laser desorption ionization-time of flight mass spectrometry (MALDI-TOF) is increasingly used in diagnostic microbiology for the routine identification of bacteria at the genus, species or subspecies level [64,65]. Generally, MALDI-TOF is prepared by coating the sample with an energy absorbent solution called matrix. The matrix is crystallized while drying and the sample within the matrix is also crystallized [66]. The sample is then subjected to a laser beam. Desorption and ionization with the laser beam produces protonated ions from analytes such as protein in the sample, which are accelerated and separated from each other based on their m/z [66,67]. Afterward, the charged analytes are detected and measured using a TOF analyzer. A characteristic peptide-mass-fingerprint (PMF) is generated for analytes in the sample based on the TOF information [66,67]. Microbial identification by MALDI-TOF is performed by comparing the PMF of unknown organism with PMFs available in the database [66].

Bacterial cultures subjected to ethanol-formic acid extraction as well as directly from bacterial colonies were used for identification [68]. Table 3 summarizes various studies carried out for the identification of *Acinetobacter* spp. using MALDI-TOF.

**Table 3.** Various studies using MALDI-TOF for the identification of *Acinetobacter* spp.

Number of specimens tested	Species identification using reference method (*rpoB* sequencing and bla*_OXA-51-like_*)	Species identified using MALDI-TOF	Accuracy rate	Study (year)
109	*A. baumannii* – 85 (bla*_OXA-51-like_*) *A. genospecies* 3 – 8,*A. berezinae* – 7 *A.calcoaceticus* – 2 *A. genospecies* 14BJ – 1 *A. genospecies* 13TU – 3 *A. junii* – 1, *A. soli* – 1 *A. beijerinkii* – 1	*A. baumannii* – 87, *A. genospecies* 3 – 19, *A. junii* – 1, *A. baylyi* – 1 *A. tjernbergiae* – 1	MALDI-TOF – at species level: Accurate identification – 51.6% Misidentification – 48.4% For *A. baumannii*: Concordance – 77.8% Sensitivity – 100% Specificity – 91.7%	Álvarez-Buylla *et al*. (2012) [76]
60	*A. baumannii* –100% (bla*_OXA-51-like_*)	*A. baumannii* – 18, *A. nosocomialis* – 18 *A. pitti* – 17 Reference strains – 7	98% of all isolates were correctly identified up to appropriate species level	Espinal *et al*. (2012) [69]
123	*A. pitti* – 42 *A. baumannii* – 22 (bla*_OXA-51-like_*) *A. nosocomialis* – 19 *A. ursingii* – 15 *A. grimontii* – 8 *A. oleivorans* – 4 *A. berezinae* – 2 *A. soli* – 2 *A. junii* – 1 *A. baylyi* – 1 *A. johnsonii* – 1 *A. radioresistens* –1 *A. genospecies* 14BJ – 1 Not reliable identification – 4	*A. pitti* – 38 *A. baumannii* – 27 *A. nosocomialis –*14 *A. ursingii* – 14 *A. junii* – 9 *A. calocoaceticus* – 3 *A. guillouiae* – 2 *A. baylyi* – 1 *A. johnsonii* – 1 *A. radioresistens* – 1 Not identified – 13	Species identification achieved for 72.4% of isolates Discordant results at species level were obtained for 13.8% of the isolates Improved sensitivity from 74.8 to 82.4% with updated database	Kishii *et al*. (2014) [72]
409 *(A. baumannii* – 24 Non-*baumannii* species – 385)	*A. pitti/ A. calcoaceticus* – 189 *A. oleivorans/A. calcoaceticus* – 6 *A. nosocomialis* – 134 *A. genomic species 33YU* – 11 *A. baumannii* – 24 *A. haemolyticus* – 2 *A. johnsonii/A. lwoffii* – 4 *A. junii/A. grimontii* – 25 *A. soli* – 9 *A. bereziniae* – 4 *A. ursingii* – 1	ACB complex – 370 *A. hemolyticus* – 2 *A. johnsonii* – 4 *A. junii* – 25 Non –*Acinetobacter* spp. – 7 *Escherichia coli* – 1	Identical results obtained with MALDI-TOF for *A. hemolyticus*, *A. johnsonii*, and *A. junii* as *rpoB* sequencing MALDI-TOF was not able to discriminate the species within the ACB complex	Wang *et al*. (2014) [24]
176	*A. baumannii* – 101 *A. nosocomialis* – 43 *A. pitti* – 10 *A. genomic species* 13BJ/14TU – 10 *A. ursingii* – 4 *A. soli* – 4 *A. bereziniae* – 2 *A. junii* – 2	*A. baumannii* – 101 *A. nosocomialis* – 43 *A. pitti* – 9 *A. ursingii* – 4 *A. junii* – 1 Mis-identification – 18	99.4% of the isolates within the ACB complex and 27.3% of the isolates among the nonbaumannii group were correctly identified with MALDI-TOF Correct ID rate – 90.3% Mis-ID rate – 0.6% No ID rate – 9.1%	Lee *et al*. (2015) [77]
Panel of ACB complex collection – 91 for confirmation of updated database – SARAMIS™ (spectral archive and microbial identification system)	*A. pitti* – 50 *A. baumannii* – 40 *A. nosocomialis* – 1	*A. pitti* – 50 *A. baumannii* – 40 *A. nosocomialis* – 1	90% of *A. baumannii* and 62% of *A. pittii* isolates were accurately identified	Pailhories *et al*. (2015) [78]
Default database – 212 isolates Improved database – 517 isolates	–	–	Default database – 69.8% concordance Improved database – 100% concordance	Jeong *et al*. (2016) [79]

ACB complex: *Acinetobacter calcoaceticus*–*Acinetobacter baumannii complex*; MALDI-TOF: Matrix-assisted laser desorption ionization-time of flight mass spectrometry.

Espinal’s group tested the previously characterized 60 *Acinetobacter* isolates (18 *A. nosocomialis*, 17 *A. pittii* and 18 *A. baumannii*) and seven reference strains of different *Acinetobacter* species using MALDI-TOF with Bruker database. It was found that 85% (n = 6) accuracy in identification at species level for the reference strains. Species-level identification was accurate and achieved for all *A. baumannii* (100%, n = 18) isolates and all *A. pittii* (94%, n = 17), except one. None of the *A. nosocomialis* were identified by MALDI-TOF as the Bruker database lacks a signature specific for *A. nosocomialis*. A set of reference spectra was created using three strains each of *A. baumannii*, *A. pittii* and *A. nosocomialis* to supplement the Bruker database. Reanalysis of spectra from all 60 isolates using a local database incorporated to MALDI-TOF was performed with reference signatures of *A. nosocomialis*, which provided unequivocal identification for all the tested strains [69].

Sedo *et al*. found an inability of MALDI-TOF to discriminate between *A. baumannii* and *A. nosocomialis* within the closely related ACB complex. In their study with a taxonomically clearly defined set of 105 strains representing the four species of the ACB complex, they evaluated a protocol with change in the matrix composition without changing the other steps of the sample processing. With the modified protocol, differentiation between *A. baumannii* and *A. nosocomialis* was improved and 13 *A. nosocomialis* strains which were wrongly assigned were correctly identified [70].

Hsueh *et al*. evaluated the ability of MALDI-TOF (Bruker Biotyper) in the correct identification of species within the ACB complex as well as species other than the ACB complex. The overall accurate identification rate of species within the ACB complex was only 85.3%.

Although the current database included *A. nosocomialis*, the correct identification rate was 72.4%. Additionally, accurate identification of non-ACB complex species like *A. junii, A. ursingii, A. johnsonii* and *A. radioresistens* was achieved using the Bruker Biotyper [71].

Kishii and colleagues evaluated MALDI-TOF for the identification of *Acinetobacter* to the species level by comparison with sequence analysis of *rpoB* using 123 isolates of *Acinetobacter* species [72]. MALDI-TOF identified 86.2% of *Acinetobacter* isolates to species level and 13% of the isolates to genus level. With *rpoB* sequence analysis, 84% were confirmed to the correct species level and discordant results were obtained for 16% isolates. The *rpoB* sequencing, which is better for species delineation, was able to assign 123 isolates to 13 species whereas MALDI-TOF assigns only to 9 species indicating the less discriminatory power of MALDI-TOF [72].

MALDI-TOF combined with chemometric tools like partial least squares discriminant analysis and hierarchical cluster analysis was reported to have (100%) accuracy in the identification within the ACB complex [73]. Overall the diagnostic efficiency of MALDI-TOF from different studies, showed 100% sensitivity, 91.7–99% specificity and 70–100% concordance with other methods for identification of *A. baumannii*.

MALDI-TOF technique was unable to identify the two novel species, *A. seifertii* and *A. dijkshoorniae* and several studies have shown that the estimated identification rate for *A. nosocomialis* range at 70% suggesting to update and improve the database for accurate identification of species within the ACB complex [68]. A total of 78 *Acinetobacter* isolates representing *A. baumannii, A. nosocomialis, A. pittii, A. dijkshoorniae* and *A. seifertii* were used to calculate the species-specific average spectra. Mean spectra derived from *A. seifertii*, *A. dijkshoorniae* and *A. nososcomialis* were added to the database [68]. Finally, when spectra from all the 78 isolates were analyzed using the custom database which included both the mean spectra from all the *Acinetobacter* species in the default taxonomy with the new reference signatures of *A. nosocomialis*, *A. seifertii* and *A. dijkshoorniae* allowed the identification of *Acinetobacter* to the correct species level within the ACB complex [68].

Variations in the sensitivity and specificity in identifying ACB complex by MALDI-TOF have been reported [68]. This is due to the methodological differences followed in each of the study. High rates of >98% of sensitivity and specificity were obtained when the bacterial colonies were subjected to a modified extraction protocol using formic acid and acetonitrile, compared with the conventional method which yielded a sensitivity and specificity of 91 and 98%, respectively [68,69]. Though both the protocols have used total mass for identification, the methodological variation of sample preparation impacts on the overall sensitivity and specificity rates.

## Conclusion

Species within the ACB complex are difficult to distinguish. The differences in clinical relevance of the ACB complex and their antimicrobial susceptibility profile warrant species-level identification. The traditional phenotypic methods such as culture and biochemical identification are slow, unreliable and less efficient at differentiating the most clinically important *A. baumannii* within the ACB complex. PCR targets such as the *bla*_OXA-51_-like gene, 16S–23S rRNA ITS, *recA* and *gyrB;* and PCR–RFLP of 16S rRNA, 16S–23S ITS and *recA* were considered as suitable markers for *Acinetobacter* speciation. However, these methods are mostly performed by reference laboratories. With an accurate database, MALDI-TOF can be helpful for species-level identification. To conclude, molecular characterization using *bla*_OXA-51_-like PCR together with *rpoB* sequencing has high discriminatory power over the conventional methods for *Acinetobacter* species identification, especially within the ACB complex.

## Future perspective

Identification of *Acinetobacter* species within the ACB complex is still challenging. Currently, available phenotypic methods are insufficient. Although various commercially available automated identification systems are widely used in clinical laboratories, such systems are still not effective for identification. Incorporation of more diverse mass spectra of different species within the ACB complex will improve the MALDI-TOF database, which eventually will be useful in accurate identification of the ACB complex at the species level. For molecular characterization, a single gene target as a biomarker is not sufficient for accurate identification of ACB complex. Whole-genome sequencing can be considered as a promising tool; however, its usage is limited in lower resource settings. Furthermore, comparative genomic studies will help us to identify the species-specific targets that can be used as a marker for molecular identification.

**Summary points**

- Identification of *Acinetobacter* species within the closely related *Acinetobacter calcoaceticus*–*Acinetobacter baumannii* complex is crucial.
- The existing phenotypic methods and commercial automated systems are not reliable for the species-level identification of *Acinetobacter*.
- At the genus level, 16S rRNA sequencing is reliable whereas *rpoB* sequencing is considered to be appropriate for species level identification.
- *bla*_OXA-51_-like gene is considered to be the species-specific biomarker for *Acinetobacter baumannii*.
- Presence of plasmid-borne insertion elements along with *bla*_OXA-51_-like gene confers carbapenem resistance, thereby limiting the use of *bla*_OXA-51_-like gene as a target for differentiating *Acinetobacter baumannii* from other *Acinetobacter* species.
- MALDI-TOF with an accurate database will be useful in the species-level identification within the *A. calcoaceticus*–*A. baumannii* complex.
